# CXCR2 Inhibition Combined with Sorafenib Improved Antitumor and Antiangiogenic Response in Preclinical Models of Ovarian Cancer

**DOI:** 10.1371/journal.pone.0139237

**Published:** 2015-09-28

**Authors:** Bharat Devapatla, Ankur Sharma, Sukyung Woo

**Affiliations:** Department of Pharmaceutical Sciences, College of Pharmacy, The University of Oklahoma Health Sciences Center, Oklahoma City, Oklahoma, United States of America; University of Bari Medical School, ITALY

## Abstract

Antiangiogenic therapy is important for the treatment of gynecological cancer. However, the therapeutic benefit derived from these treatments is transient, predominantly due to the selective activation of compensatory proangiogenic pathways that lead to rapid development of resistance. We aimed to identify and target potential alternative signaling to anti-vascular endothelial growth factor (VEGF) therapy, with a view toward developing a combination of antiangiogenic agents to provide extended therapeutic benefits. We developed a preclinical *in vivo* phenotypic resistance model of ovarian cancer resistant to antiangiogenic therapy. We measured dynamic changes in secreted chemokines and angiogenic signaling in tumors and plasma in response to anti-VEGF treatment, as tumors advanced from the initial responsive phase to progressive disease. In tumors that progressed following sorafenib treatment, gene and protein expression levels of proangiogenic CXC chemokines and their receptors were significantly elevated, compared with responsive tumors. The chemokine (C-X-C motif) ligand 8 (CXCL8), also known as interleukin-8 (IL-8) increase was time-dependent and coincided with the dynamics of tumor progression. We used SB225002, a pharmacological inhibitor of chemokine (C-X-C motif) receptor 2 (CXCR2), to disrupt the CXC chemokine-mediated functions of ovarian cancer cells in *in vitro* assays of cell growth inhibition, spheroid formation, and cell migration. The combination of CXCR2 inhibitor with sorafenib led to a synergistic inhibition of cell growth *in vitro*, and further stabilized tumor progression following sorafenib *in vivo*. Our results suggest that CXCR2-mediated chemokines may represent an important compensatory pathway that promotes resistance to antiangiogenic therapy in ovarian cancer. Thus, simultaneous blockage of this proangiogenic cytokine pathway using CXCR2 inhibitors and the VEGF receptor (VEGFR) pathway could improve the outcomes of antiangiogenic therapy.

## Introduction

Ovarian cancer is the leading cause of death from gynecological cancers in the U.S. When treated with current standard-of-care chemotherapy, the five-year survival rate of advanced disease remains low. Ovarian tumors are richly vascularized; a correlation exists between microvascular count and biological aggressiveness [1, 2]. Angiogenesis plays a central role in both normal ovarian function and the development and progression of ovarian cancer [3]. Tumor angiogenesis is critical to ascites development and ovarian cancer metastasis into the peritoneal space [4]. As angiogenesis is a critical step in the propagation of malignant tumor growth and metastasis [5], angiogenesis is a valid target in the treatment of ovarian cancer [6]. Multiple proangiogenic factors promote the process of vessel formation with VEGF as a central player in tumor mediated angiogenesis [5]. A number of antiangiogenic agents, including the therapeutic antibody against VEGF bevacizumab and multi-targeted kinase inhibitors of VEGF receptors (VEGFR), have been actively investigated or are in development as treatments for advanced disease. Treatment with bevacizumab provided a progression free survival (PFS) benefit in advanced ovarian cancer when administered in combination with chemotherapy [7, 8]. Several VEGFR-targeting multi-kinase inhibitors such as nintedanib, trebananib, pazopanib, sunitinib, sorafenib, cediranib have been tested in various phases of ovarian cancer clinical trials [9]. Some of these agents, including pazopanib, nintedanib, cediranib, and trebananib, have been evaluated in randomized Phase III clinical trials, and all have demonstrated a progression-free survival (PFS) benefit [10]. However, the therapeutic benefit of anti-VEGF therapy is short-lived. Tumor progression is eventually restored after an initial response, indicating emerging resistance. Understanding the mechanisms of tumor escape from anti-VEGF therapy is critical to finding strategies to circumvent resistance. Several mechanisms are involved in acquired resistance to anti-VEGF therapy, including activation of alternative pathways to circumvent VEGF inhibition, thereby resuming tumor angiogenesis and tumor growth [11]. Thus, combining different antiangiogenic therapies may be a strategy to provide lasting therapeutic benefits.

Many growth factors that are involved in ovarian cancer invasion are also prominent in its angiogenesis [4]. Inflammatory chemokines play an important role in the angiogenesis and progression of ovarian cancer. The interactions between chemokines produced by the ovarian cancer cells and chemokine receptors expressed by endothelial cells and the tumor microenvironment promote tumor growth by stimulating angiogenesis and increasing migration, invasion, and cell proliferation [12–14]. Among the chemokine subsets, the CXC chemokines with the 3-amino acid (Glu-Leu-Arg/ELR) motif (ELR^+^), including CXCL1, 2, and 3 (GRO-α,β and γ), CXCL5, CXCL6, CXCL7, and CXCL8 (IL-8), are potent regulators of angiogenesis and mediate their angiogenic activity through the chemokine receptor CXCR2 [14]. Overexpression of the proangiogenic chemokines and CXCR2 was correlated with poor prognosis in patients with ovarian cancer [12, 15–17]. Thus, ELR^+^CXC proangiogenic chemokine pathways are a potential alternative to promoting angiogenesis in ovarian tumors.

To identify effective strategies to circumvent the mechanism by which ovarian cancer bypasses anti-VEGF therapy, we generated a xenograft model of antiangiogenic therapy resistance that closely mimics clinical resistance to ovarian cancer. We identified changes in cytokine and angiogenic pathways in tumors resistant to antiangiogenic therapies, as tumors progressed from the initial responsive phase to the refractory phase. Our *in vitro* and *in vivo* results indicated that co-targeting the CXCR2 proangiogenic cytokine axis with anti-VEGF inhibition is an effective strategy to provide extended therapeutic benefits in pre-clinical models of ovarian cancer.

## Materials and Methods

### Cells and reagents

The SKOV-3 ovarian cancer cell line was obtained from the American Type Culture Collection. A2780 and OVCAR429 ovarian cancer cells were kindly provided by Dr. Danny Dhanasekharan (Stephenson Cancer Center, OUHSC). The A2780 ovarian cancer cell line was initially obtained from Sigma-Aldrich (St. Louis, MO). OVCAR429 are ovarian cancer cells that have been previously published [18, 19]. A2780 and OVCAR429 cells were maintained in RPMI medium (Invitrogen). SKOV-3 cells were maintained in McCoy’s 5A medium (Invitrogen). Media were supplemented with 10% fetal bovine serum (Invitrogen), 100 IU/mL of penicillin, and 100 μg/mL of streptomycin (Invitrogen) at 37°C in a humidified incubator containing 5% CO_2_. Human umbilical vein endothelial cells (HUVECs) and endothelial cell media were purchased from Cell Applications (San Diego, CA). Sorafenib was obtained from LC laboratories (Woburn, MA). SB225002 (*N*-(2-hydroxy-4-nitrophenyl)-*N*′-(2-bromophenyl)urea) is a potent and selective non-peptide inhibitor of CXCR2 (IL-8R) chemokine receptor. SB225002 was procured from Tocris Bioscience (Bristol, UK). Drug preparations for sorafenib and SB225002 were performed according to methods described elsewhere [20, 21].

### Animals and drug treatment

Six-week-old female athymic *nu/nu* nude mice were purchased from Charles River Laboratories, Inc., through NCI (Frederick, MD). All procedures involving mice were carried out in accordance with the guidelines of the Institutional Animal Care and Use Committee (IACUC), and the protocol was approved by the University of Oklahoma Health Sciences Center (OUHSC) Institutional Animal Care and Use Committee (Protocol Number: 12-154-H). Mice were given subcutaneous injections of 5 × 10^6^ SKOV-3 cells in the right flank. Tumor size was measured twice weekly using digital calipers (Mitutoyo) with an accuracy of ± 0.02 mm. Tumor volume was calculated as 4/3 π length x width x height. Mice were treated with saline or sorafenib when tumors reached approximately 80 mm^3^ in volume, 32 days after tumor cell implantation. Sorafenib was administered daily by oral gavage at a dose of 30 mg/kg. Treatment continued until tumors grew to 20 mm (the maximum growth allowable by IACUC), at which point the mice were euthanized. Xenograft tumors that increased less than 50% of the initial tumor volume at the start of treatment were considered treatment-responsive, as this showed a long-term trend toward tumor stasis [22]. Tumors that progressed with a long-term trend toward continued growth after an initial response to treatment were considered to display emerging phenotypic treatment-resistance. At various time points, we used retro-orbital puncture to collect about 30 μl of blood into EDTA-containing tubes to determine the time profiles of circulating cytokines and angiogenic factors. The animals were anesthetized prior to the retro-orbital blood collection using 2% isoflurane in an inhalation chamber regulated with a calibrated vaporizer. The mice were monitored daily and euthanized as soon as there was any evidence that the mouse was in pain from the tumor or drugs or if the tumor burden reached 20 mm. The early euthanasia endpoints include moderate or severe toxicity, including rapid weight loss of greater than 10% of body weight, gradual weight loss of greater than 15%, weakness, non-responsiveness, respiratory difficulties, severe abnormal neurological signs, bleeding, trauma or the inability to eat or drink. After the eight weeks of drug treatment, all of the mice were euthanized using CO_2_ asphyxiation and necropsied. Blood and tumor tissues were collected for the analyses described below. Plasma was isolated and stored at -80°C until analysis.

For the *in vivo* combination study, SKOV-3 xenografts were treated with 30 mg/kg/day sorafenib until the emergence of phenotypic resistance as defined above. Sorafenib-resistant animals were randomized into three groups to receive sorafenib, SB225002, or combination of sorafenib and SB225002. SB225002 was administered once daily by intra peritoneal (IP) injection at a dose of 10 mg/kg. Combination treatment group daily received an oral dose of 30 mg/kg sorafenib plus 10 mg/kg SB225002 (IP) until the end of the study.

### Cytokine and angiogenic growth factor multiplex assay

We performed a multiplex assay to identify the changes in circulating levels of angiogenic growth factors in treatment-sensitive and-resistant animals. Plasma concentrations of tumor- and stroma/host-emanating angiogenic growth proteins were separately determined using human (Millipore, cat # HAGP1MAG-12K) and mouse (Millipore, cat # MAGPMAG-24K) angiogenesis/growth factor magnetic bead panels, respectively. A total of 17 human and 24 mouse soluble angiogenic factors were quantified. A list of quantified analytes is provided in the supporting methods (S1 File). The soluble levels of CXC chemokines CXCL1, 2, 5, and 6 in mouse plasma samples were determined via a multiplex assay (Bio-Rad, cat # 171- AK99MR2).

### Growth inhibition assay

*In vitro* cell growth inhibition was measured via MTS assay (Promega). Briefly, 5 x 10^3^ HUVEC or SKOV-3 cells were seeded into 96-well plates and incubated overnight. We added various concentrations ranging from 0.01 to 100 μM of sorafenib or SB225002 to 100 μl fresh medium and incubated the cells for the indicated time. We added 20 μl of CellTiter 96 AQueous One solution reagent and incubated the cells for 1 hour. Absorbance was measured at 450 nm. Cell viability was calculated relative to controls (cells treated with vehicle alone). Averages of three replicates per sample were used for analysis.

The combined effects of sorafenib and SB225002 were determined using various ovarian cancer cells and HUVECs. IC_50_ values were determined for both drugs with each cell line. The top concentration of the combination was prepared by mixing the sorafenib and SB225002 at 1:2 ratio of their IC_50_ for ovarian cancer cells and 1:1 ratio for HUVECs based on their IC_50_ values. Serial dilution (3x) was carried out from the top concentration to obtain a total of 5 dilutions (*n* = 3 replicates). The cells (5000 cells/well) were then incubated at 37°C for 48 hours. Cell survival was studied using MTS assay as described above. The nature of the combinatory effects of the two drugs was determined by calculating the combination index (CI) values, which characterizes for synergy (CI < 1), additivity (CI = 1), or antagonism (CI > 1), using CalcuSyn (Biosoft, Cambridge, UK).

### Angiogenesis tube formation assay

Growth factor-reduced Matrigel matrix (Corning) was thawed at 4°C overnight and then bottom coated in a 96-well plate (50 μl per well) at 37°C for 30 minutes. Next, 100 μl of media containing HUVECs (10,000 cells), with or without sorafenib, and SB225002 was added to each well on top of the solidified Matrigel matrix and incubated at 37°C for 16 hours. Wells were fixed in 4% paraformaldehyde and networks were imaged using a phase-contrast microscope. For the quantification of networks, WimTube software was used (Wimasis).

### Transwell migration assay

Cell migration assays were performed using 8-μm-pore transwell inserts (Corning) as previously described [23]. To the lower chambers, 500 μl of endothelial growth media was added. Aliquots of 2 × 10^4^ HUVECs in 200 μl of endothelial basal medium were seeded into the upper chambers. Both upper and lower chambers contained sorafenib or SB225002 at the indicated concentrations. After the assay ran for 24 hours at 37°C, non-migrated cells were removed from the upper surface of the filter using cotton swabs. Cells on the lower surface of the membrane were fixed with ice-cold methanol and stained with crystal violet. Cells were counted under an optical microscope (×40).

### Spheroid assay

SKOV-3 cell spheroids were grown using the liquid overlay method [24]. Cells were plated in agarose-coated 96-well plates containing 200 μl of medium (2000 cells/well). Cells were incubated for 72 hours until spheroids formed. Cells were treated with the indicated concentrations of sorafenib or SB225002. Every other day, we replaced 100 μl of old medium with fresh medium and drugs. Spheroids were monitored for up to 10 days. The sizes of spheroids were measured using ImageJ software (http://imagej.nih.gov/ij/).

### Immunohistochemistry

Immunohistochemistry (IHC) data quantification is described in the supporting methods (S1 File). We used antibodies specific to CD-31 (Abcam, cat#ab28364) and Ki-67 (Abcam, cat#ab16667) to determine microvessel density and tumor cell proliferation, respectively. A total CXCR2 expression in tumor and stroma cells was determined in mouse xenograft tumors using anti-CXCR2 antibody (Abcam, cat#ab14935).

### Gene expression of ELR^+^ CXC chemokines

Transcriptome sequencing was performed using the Illumina MiSeq sequencer and Illumina TruSeq RNA v2 sample preparation kit and protocols (Illumina, Inc.). Sample preparation and library construction is described in the supporting methods (S1 File).

### Statistical analysis

Statistical analyses were performed with mean ± standard deviation (S.D.) values using Student’s t-test, unless otherwise stated. Statistical significance was set at *P* < 0.05. Drug combination data was analyzed by Calcusyn software using the median-effect method by Chou and Talalay [25]. Immunohistochemistry data was analyzed using ImageJ software. Brief methodologies are given in the supporting data. We used GeneSifter software (Geospiza, Inc.) to analyze data from our studies of gene expression in xenograft tumors.

## Results

### *In vivo* phenotypic tumor resistance to antiangiogenic treatment

**Fig 1:**
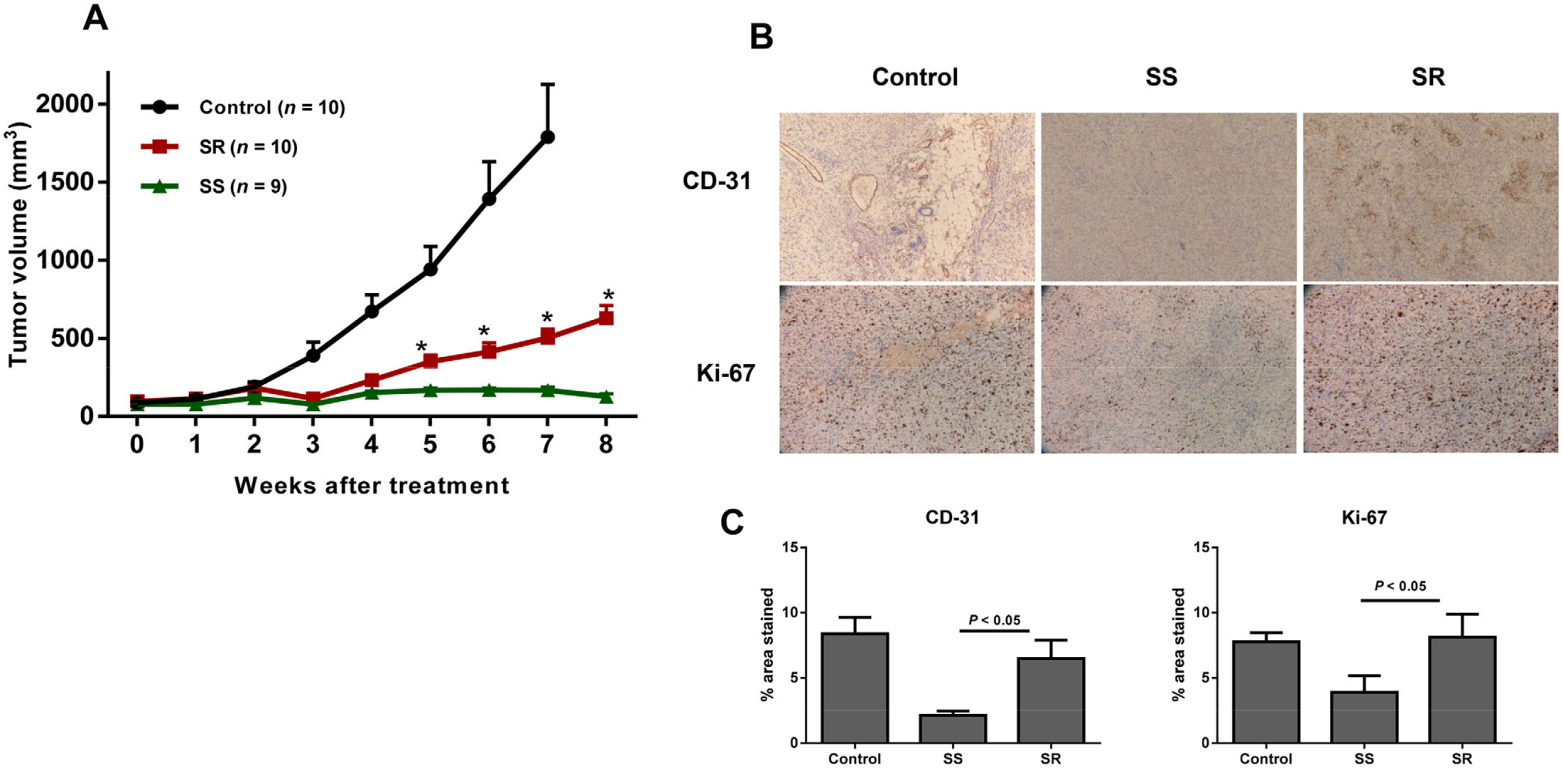
Phenotypic resistance to anti-VEGFR therapy in a SKOV-3 ovarian cancer xenograft mouse model. **A)** SKOV-3 xenografts were treated for eight weeks with 30 mg/kg sorafenib via daily oral gavage. Controls were treated with the treatment vehicle alone. Tumor volumes were measured twice a week and are represented as mm^3^ ± SEM. Out of 19 mice, 10 developed resistance to the sorafenib treatment. Black, red, and green lines indicate the mean tumor volume progressions of control, SR, and SS mice, respectively. A significant difference in tumor volume (* *P* < 0.05) was observed between the treatment-resistant and-sensitive groups, starting at week five of treatment. SS = sorafenib-sensitive; and SR = sorafenib-resistant. B) Representative immunostaining for CD-31 (top row), and Ki-67 (bottom row) in control, sorafenib-sensitive (SS), and sorafenib—resistant (SR) tumors. C) Vessel density (CD-31) and proliferation index (Ki-67) were significantly increased in sorafenib-resistant tumors compared with the sorafenib-sensitive tumors.

We observed tumor growth inhibition for up to three weeks of treatment with sorafenib (Fig 1A). After this initial responsive period, some tumors began progressing, as evidenced by increased tumor size despite continued treatment. Over the two-month treatment period, 10 of 19 (53%) xenograft mice showed tumor progression and regrowth. None of the mice became ill or dead prior to the experimental end point. Data from IHC staining revealed the typical antiangiogenic-resistant signature in tumor tissues (Fig 1B). Tumor vessel density (CD-31), and cell proliferation (Ki-67) were significantly higher (2–4 fold, *P* < 0.05) in tumors that progressed from the treatment than in responsive tumors (Fig 1C), suggesting that the progressive tumors were able to resume angiogenesis and thereby tumor regrowth.

### Dynamic changes of circulating angiogenic factors in response to anti-VEGF treatment

To identify the changes in secreted angiogenic and cytokine signaling, we compared circulating angiogenic factor concentrations in plasma samples obtained from treatment-sensitive and-resistant mice. Samples were obtained during the responsive (2–3 weeks after treatment) and refractory phases (6–8 weeks after treatment). Because tumor vasculature in xenografts is formed by mouse endothelial progenitor cells, we used multiplex angiogenic panels for both mice and humans, to distinguish the origin.

**Fig 2:**
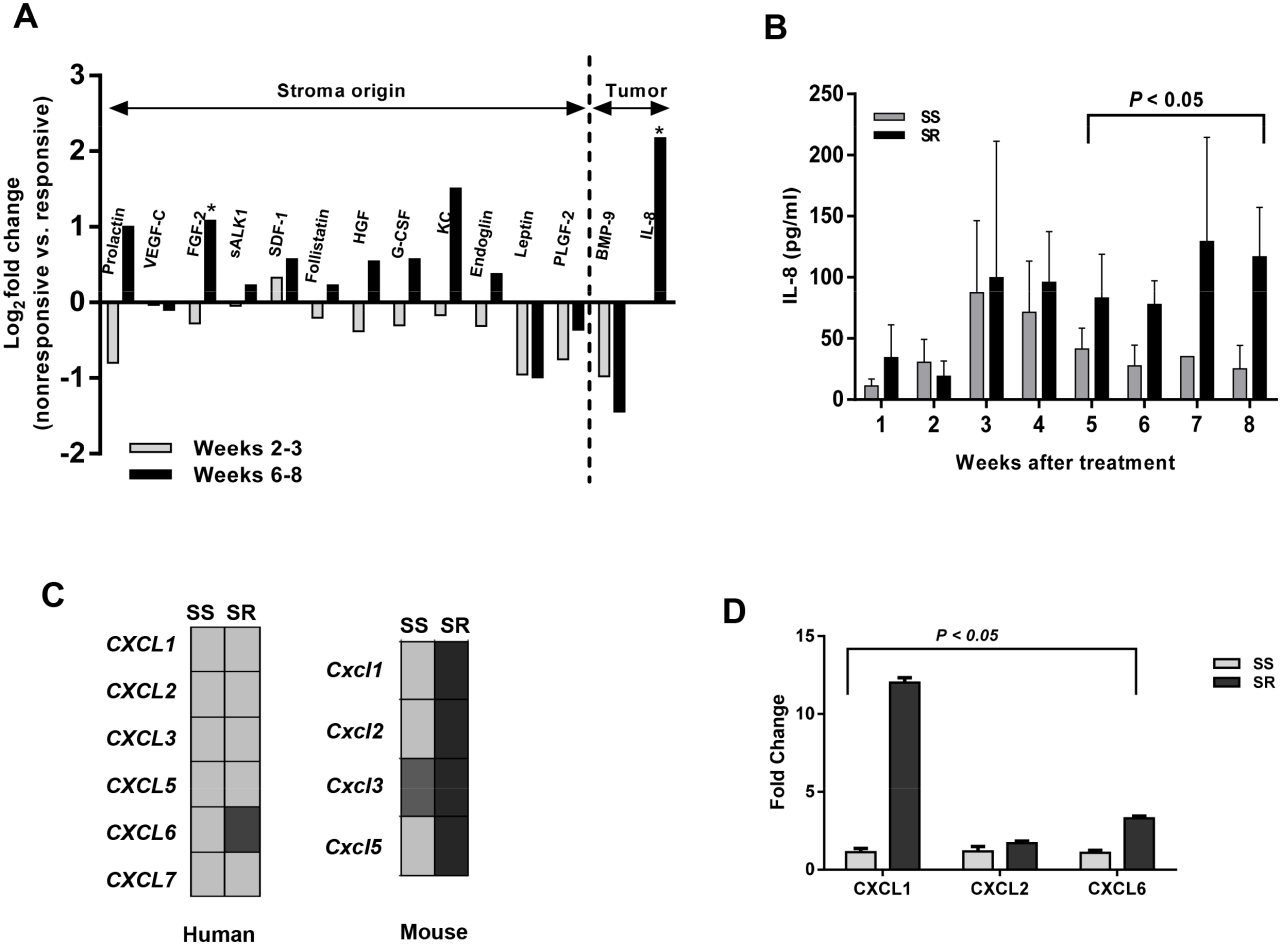
Dynamic changes in circulating angiogenic factors and gene expression of ELR^+^ *CXC* chemokines in response to anti-VEGFR treatment. A) Fold changes in circulating angiogenic factors between treatment-resistant and-sensitive groups during the responsive (2–3 weeks) and resistant (6–8 weeks) phases in mice treated with sorafenib. Both mouse (stroma) and human (tumor cell) soluble angiogenic factors were quantified by multiplex assay. Only angiogenic factors that were detected from both mouse and human arrays are represented as Log_2_ fold changes of treatment-resistant versus treatment-sensitive xenografts. Significant (*P* < 0.05) changes between responsive and resistant phases are indicated by asterisk (*). B) Time-dependent comparison of plasma IL-8 concentration (pg/ml ± S.D.) between treatment-resistant (SR) and—sensitive (SS) groups of mice treated with sorafenib. Time profiles of plasma IL-8 were in accordance with tumor progression of phenotypic-resistant and-sensitive tumors. During treatment, plasma was collected weekly by the staggered sampling method, and IL-8 levels were measured by multiplex assay. C) Illumina sequencing analysis of human and mouse ELR^+^ *CXC* chemokine gene expression. Human ELR^+^ *CXC* chemokine gene expression analysis showed that *CXCL6* expression was significantly higher in the resistant group (2.5 fold higher, *P* < 0.05) than in the sensitive group. No significant difference was observed between sorafenib-sensitive and-resistant groups for CXCL1, 2, 3, 4, 5, and 7 expression levels. Mouse ELR^+^ *CXC* chemokine gene expression analysis showed that all indicated cytokines, except *Cxcl2*, were significantly higher in resistant groups (> 2 folds, *P* < 0.05) than the sensitive groups. SS = sorafenib-sensitive; and SR = sorafenib-resistant. D) CXC chemokine plasma concentrations (pg/ml) in SKOV-3 xenograft mice. CXCL1, CXCL2, and CXCL6 levels were significantly (*P* < 0.05) higher in sorafenib-resistant groups than in sorafenib-sensitive groups.

During the first three weeks of treatment, there were no significant differences between the concentrations of angiogenic proteins in any groups (Fig 2A). When phenotypic resistance emerged 6–8 weeks post treatment, angiogenic factor differences were evident between groups. Various mouse-originated angiogenic factors, including fibroblast growth factor (FGF)-2, hepatocyte growth factor (HGF), KC, and prolactin, were upregulated in the sorafenib-resistant group, but not in the sorafenib-sensitive group (Fig 2A). During the refractory phase, tumor-secreted CXCL8 (IL-8) levels increased > 4 folds (*P* < 0.05) in the sorafenib-resistant tumors.

We also quantified IL-8 levels weekly for up to eight weeks of sorafenib treatment. For up to 4 weeks of sorafenib administration, we found no significant differences in IL-8 level between the treatment-sensitive and-resistant groups (Fig 2B). Starting at week five, significantly elevated (*P* < 0.05) levels of IL-8 were noted in sorafenib-resistant tumors. These results indicate that IL-8 levels dynamically changed over time, coinciding with the time profile of tumor progression. In addition to IL-8, data from our gene expression study (Fig 2C) and cytokine multiplex assay (Fig 2D) showed that expression of other ELR^+^CXC chemokines (e.g., CXCL1, -2, -5, and -6) was high in treatment-resistant tumors, compared with their counterparts.

### CXCR2 expression in ovarian tumors

**Fig 3:**
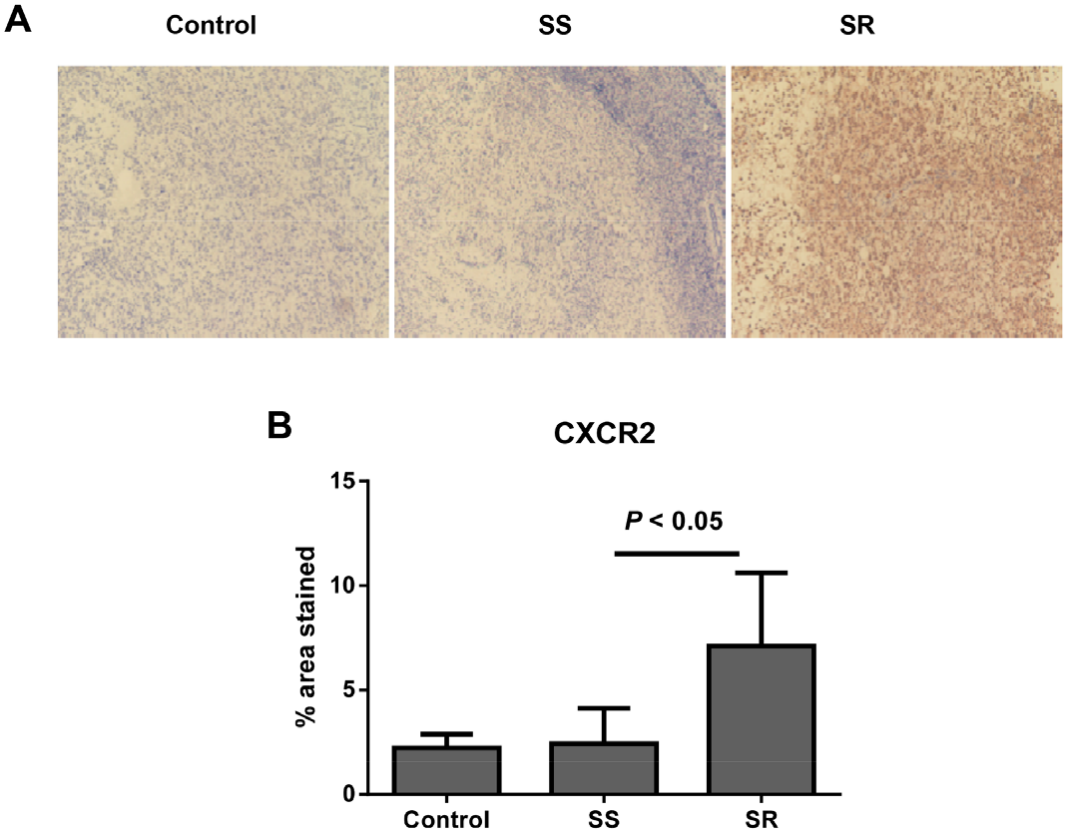
CXCR2 expression in SKOV-3 ovarian xenograft tumors. A) Representative immunostaining for CXCR2 in control, sorafenib-sensitive (SS), and sorafenib—resistant (SR) tumors. B) CXCR2 expression was elevated in sorafenib-resistant tumors (*P* < 0.05) compared with sorafenib-sensitive tumors.

We performed CXCR2 staining in xenograft tumors of sorafenib treatments to compare its expression (Fig 3A) in sensitive and resistant groups. CXCR2 expression was elevated in resistant tumors compared with sensitive and control tumors. We observed a threefold increase in resistant tumors compared with sensitive tumors (*P* < 0.05, Fig 3B) in the sorafenib treatment group.

### *In vitro* synergistic effects of sorafenib and SB225002

**Fig 4:**
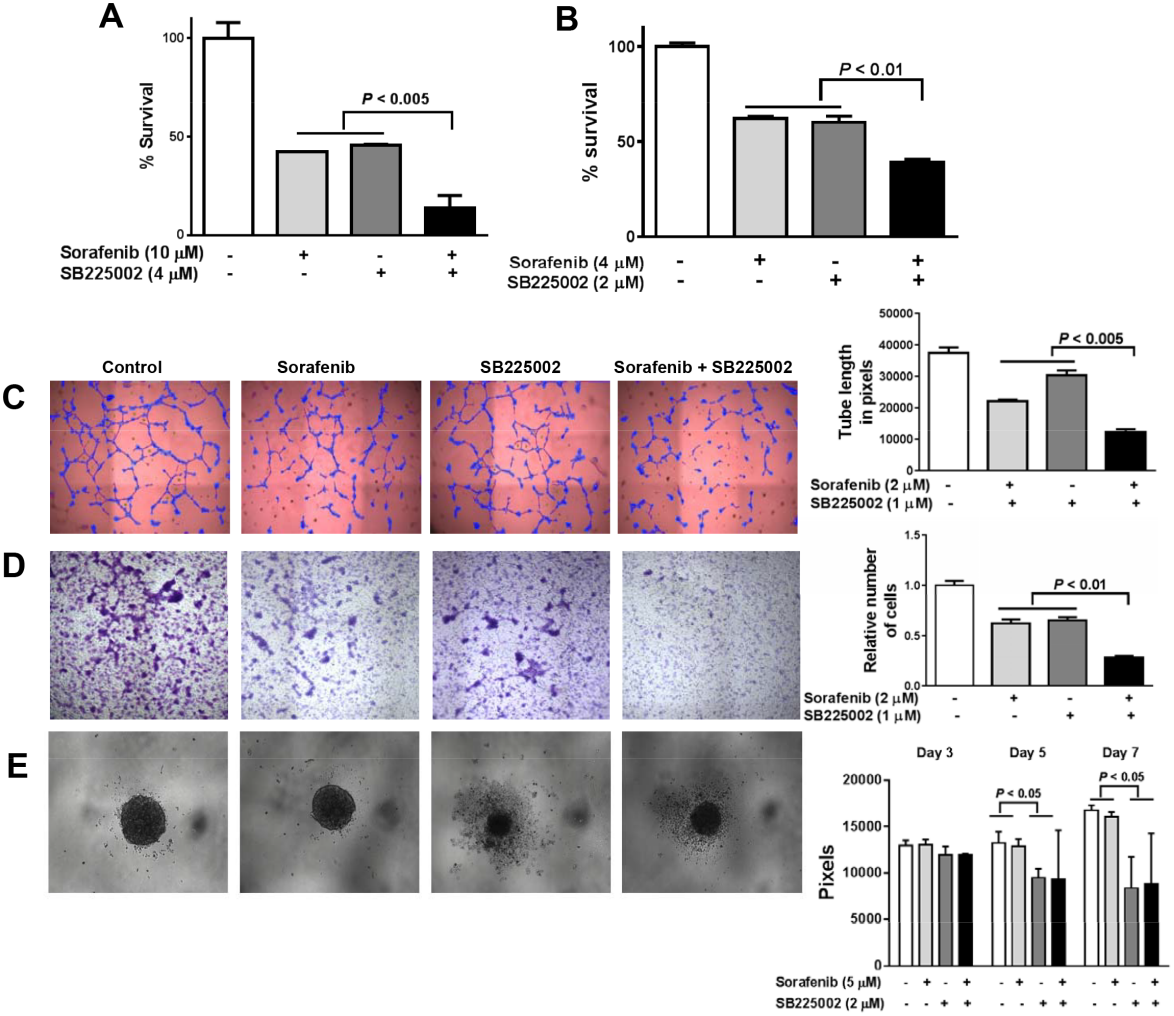
*In vitro* effects of combined SB225002 and sorafenib. SB225002 significantly enhanced the growth inhibition effects of sorafenib on SKOV-3 (A) and HUVEC (B) cells. A) A combination of 10 μM sorafenib and 4 μM SB225002 synergistically inhibited the growth of SKOV-3 cells, compared with each drug alone (*P* < 0.005). B) A combination of 4 μM sorafenib and 2 μM SB225002 synergistically inhibited the growth of HUVEC cells, compared with each drug alone (*P* < 0.01). C) Representative images of tube formation assay of HUVECs treated with or without 1 μM SB225002 or 2 μM sorafenib. Tube formation was analyzed using Wimtube analysis software and tube length is presented in pixels. Addition of SB225002 to sorafenib treatment induced a statistically significant decrease in HUVECs tube formation (*P* < 0.005). Data are shown as mean tube length in pixels ± S.D. of three independent experiments. D) Representative images of HUVECs migration assay using transwell chamber. To quantitate migratory cells, three independent fields of migratory cells per well were photographed under the phase contrast microscope. The number of cells per field was counted and averaged. Cell counts are expressed as relative number of cells migrated to lower side of the transwell chamber with the results from control cells given as 1. The inhibitory effect of sorafenib (2 μM) on migration was significantly enhanced by 1 μM SB225002 (*P* < 0.01). E) Spheroidal growth is impaired by CXCR2 inhibition in SKOV-3 cells. Spheroid areas were measured using ImageJ software. The values represented on the y axis are pixels ± S.D. of three independent experiments.

Based on the above findings of the upregulation of CXCR2 expression and multiple ELR^+^CXC proangiogenic cytokines that mediate their angiogenic effects via CXCR2 receptor, we investigated CXCR2-mediated cytokine effects using SB225002, a small molecule inhibitor of chemokine receptor CXCR2 (IL-8R), in combination with sorafenib *in vitro*. We chose CXCR2 receptor inhibitors over anti-CXC chemokine antibodies to avoid the redundant function of ELR^+^CXC chemokine signaling. The combination of sorafenib and SB225002 produced significantly higher growth inhibition (*P* < 0.005) in tumor cells and HUVECs than did either treatment alone (Fig 4A and 4B). The CI values at different concentration ratios in SKOV-3 (0.32–0.61) and HUVECs (0.87–1.1) indicated the synergy or additivity between SB225002 and sorafenib. IC_50_ values of sorafenib and SB225002 in the tested ovarian cancer cell lines and HUVECs are provided in the supporting data (S1 Table). Concentration-effect curves of the combination treatment compared with sorafenib alone are shown in the S1 Fig.

Endothelial tube formation and migration are important characteristics of tumor angiogenesis. We performed transwell chamber migration assays and matrigel-based tube formation assays using HUVECs to evaluate the antiangiogenic potential of sorafenib and SB225002 in combination. As shown in Fig 4C, the sorafenib (2 μM) and SB225002 (1 μM) combination treatment almost completely suppressed HUVEC tube formation. The combination of sorafenib and SB225002 significantly inhibited endothelial tube length compared with sorafenib (≥ 2 fold, *P* < 0.005) or SB225002 (≥ 3 fold, *P* < 0.005) alone. In addition, results from the transwell migration assay demonstrated that SB225002 significantly potentiated the sorafenib-mediated inhibition of endothelial migration (Fig 4D). This combination caused an approximately twofold decrease of HUVECs migration when compared with sorafenib and SB225002 treatments alone (*P* < 0.01). Altogether, these results indicate that combination treatment of sorafenib and SB225002 could significantly impair the angiogenic potential of HUVECs *in vitro*.

Since IL-8 plays a role in tumor cell aggregation, we performed a spheroid assay to study the effects of sorafenib and SB225002 on spheroid growth. SB225002 disrupted formation of SKOV-3 spheroids at a 2 μM concentration: sorafenib did not (Fig 4E). A combination of sorafenib and SB225002 reduced the growth of spheroids (Fig 4E).

### SB225002 and sorafenib combination therapy in sorafenib-resistant xenograft mice

**Fig 5:**
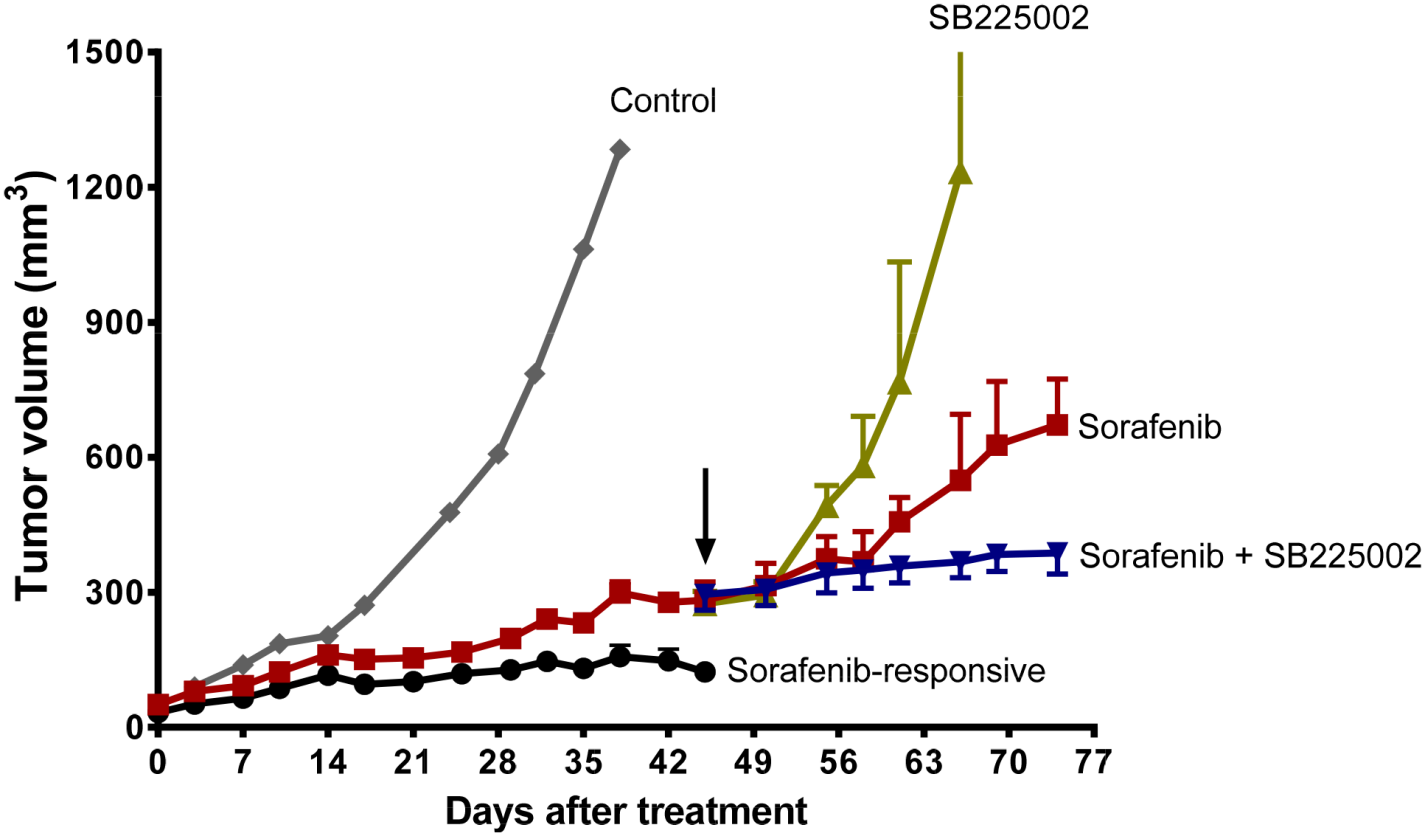
CXCR2 inhibition *in vivo* stabilizes progression of sorafenib-resistant tumors. SKOV-3 xenografts were treated with a daily dose of 30 mg/kg sorafenib until tumors developed phenotypic resistance to the treatment (~46 days). Mice with resistant tumors were then randomized into groups to receive one of the following: 30 mg/kg sorafenib alone (*n* = 6), 10 mg/kg SB225002 alone (*n* = 6), or sorafenib plus SB225002 (*n* = 6). The arrow indicates the start of treatment on day 46. Tumor volume was measured at the indicated times and is presented as mean ± S.D. The sorafenib and SB225002 combination led to 42% reduction in tumor growth compared with sorafenib alone (*P* < 0.05).

We further evaluated the effects of combined treatment in sorafenib-resistant tumor xenografts *in vivo*. We treated mice with sorafenib until the xenograft tumors developed resistance. After 46 days of sorafenib treatment, mice with sorafenib-resistant tumors received sorafenib, SB225002, or a combination of the two drugs. Tumors treated with sorafenib alone progressed, but did so more slowly than the control tumors (Fig 5). Tumor progression was significantly suppressed when SB225002 was added to sorafenib (Fig 5). At the end of treatment, the tumors from mice that received the combination treatment were 42% smaller in volume than tumors from mice that received sorafenib alone. Interestingly, treatment with SB225002 alone did not inhibit tumor growth that progressed from sorafenib treatment, suggesting the need for continued blockage of VEGF signaling pathway. Our results show that the combined CXCR2 inhibition with sorafenib effectively alleviated tumor progression and provided extended therapeutic benefit.

## Discussion

Angiogenesis plays an important role in the development and progression of ovarian cancer. Ovarian tumors are richly vascularized, and a high degree of tumor angiogenesis in ovarian tumors predicts poor clinical outcome [1, 2, 26]. Ovarian tumors overexpress proangiogenic factors, including VEGFs, FGFs, angiopoietin, platelet-derived growth factors (PDGFs), and several pro-angiogenic cytokines [10]. To date, encouraging results have been obtained with ovarian cancer trials incorporating VEGF-pathway inhibitors, the therapeutic antibody bevacizumab, and small molecule receptor tyrosine kinase inhibitors (TKIs)[27]. Adding bevacizumab to chemotherapy reduced the risk of disease worsening and/or death by 62% when compared with chemotherapy alone [8, 28]. This finding led to the recent FDA approval of bevacizumab in combination with chemotherapy in recurrent disease. Recent phase III clinical studies also showed that VEGFR TKIs significantly increase PFS when used as a maintenance therapy [27, 29], suggesting that an antiangiogenic strategy is a valid and important treatment option for ovarian cancer. However, the long-term benefit of anti-VEGF therapy has consistently been limited by rapid development of resistance. This approach has failed to provide overall survival benefits for patients with ovarian carcinoma, beyond improved PFS. Thus, effective strategies to delay or mitigate tumor escape after anti-VEGF therapies are critically needed. To explore this problem, we established an ovarian cancer xenograft model that mimics treatment resistance to the antiangiogenic drug sorafenib.

We found that circulating levels of ELR^+^ CXC chemokines, including CXCL1, CXCL2, CXCL3, CXCL5, and CXCL8, are highly elevated in mice with tumors that have progressed following sorafenib treatment, compared with mice with stabilized tumors that remained responsive to the therapy. CXCL8/IL-8 is a prototype of the ELR^+^ CXC chemokines that promote angiogenesis, tumorigenesis, and metastasis in many human cancers, including ovarian cancer [30–32]. Elevated IL-8 was detected in ovarian cyst fluid, ascites, serum, and tumor tissue from ovarian cancer patients; increased IL-8 expression was correlated with poor prognosis and chemo-sensitivity [15]. High levels of IL-8 in ovarian cancer patients are associated with disease-specific mortality [33]. In addition, CXCL1, CXCL2, and CXCL5 (ENA-78) can stimulate cell proliferation and migration in ovarian cancer [34, 35]. Our data showed that IL-8 levels increased in parallel to the emerging refractory phase. This dynamic increase in IL-8 levels in treatment-resistant tumors suggests that IL-8 plays a significant role in conferring the acquired resistance to sorafenib treatment. The changes in some of angiogenic growth factors may be associated with tumor burden. Among CXCR2-mediated cytokines, we found some CXCLs increased more than tumor increase and some were associated with tumor size. However, it is also important to note that tumor compositions emanating angiogenic factors may not be proportional to the tumor size and long-term drug treatment may further change their distribution. Thus, the normalization only to tumor size may not be appropriate for some growth factors. Nonetheless, consistent with our results, other studies have shown that IL-8 mediates acquired resistance to another VEGFR TKI, sunitinib, in preclinical models of renal cell carcinoma; neutralizing IL-8 with anti-IL-8 antibody reversed the resistance to sunitinib [22]. Similarly, IL-8 was found to modulate intrinsic resistance to bevacizumab in squamous cell carcinoma of the head and neck [36]. Taken together, these findings emphasize the global role of IL-8 in conferring resistance against the class of drugs that targets VEGF pathways.

The ELR^+^CXC chemokines mediate their angiogenic effects through two chemokine receptors, CXCR1 and CXCR2 [37–40]. Two CXCLs (6 and 8) are CXCR1 ligands, whereas all seven ELR^+^CXC chemokines are CXCR2 ligands. CXCR1 and 2 are expressed in cancer cells, endothelial cells, infiltrating neutrophils, and tumor-associated macrophages, suggesting their important regulatory roles within the tumor microenvironment [41]. CXCR2 controls ovarian tumorigenesis by modulating the signaling network comprising PI3K/AKT, NF-κB, MAPK, and STAT3, which regulates cell cycle progression, apoptosis/anti-apoptosis, and angiogenesis [16, 42]. Studies have demonstrated the inhibition of endothelial cell migration by neutralizing CXCR2 antibody [43] and delaying vascularization in CXCR2 knockout mice [44]. CXCR2 is overexpressed in ovarian tumors [16]; its overexpression is associated with poor survival for patients with ovarian cancer [16, 17]. In addition to the increased gene and protein expressions of multiple ELR^+^CXC chemokines, our immunohistochemistry data (Fig 3) showed that CXCR2 is overexpressed in anti-VEGF treatment-resistant tumors, compared with treatment-sensitive tumors. Such a signaling redundancy in these CXC chemokines and their receptors should be a significant consideration in designing therapeutic strategies to inhibit the angiogenic effects of CXC-chemokine signaling. CXCR2 is the only CXC chemokine receptor mediating ELR^+^CXC chemokine-induced angiogenesis [45]. Given the functional redundancy in ELR^+^CXC chemokine signaling, we hypothesized that targeting CXCR2 receptors will more effectively inhibit the proangiogenic chemokine pathways than will agents blocking the individual CXC ligands (e.g., therapeutic antibody).

We used CXCR2 inhibitor SB225002 to target ELR^+^CXC chemokine-mediated effects in our *in vitro* and *in vivo* studies. We also reasoned that using VEGFR inhibitors would be more effective when combined with the CXCR2 inhibitor than the anti-VEGF antibody, because IL-8 can stimulate VEGFR2 phosphorylation in a VEGF-independent manner, as a study recently demonstrated [46]. Our results show that sorafenib and SB225002 synergistically inhibited the growth of various ovarian cancer cells and HUVECs. As endothelial tube formation and migration are important steps of angiogenesis, we investigated the inhibitory effect of sorafenib and SB225002 on these processes. Our data indicates that combination treatment of sorafenib and SB225002 could significantly impair the angiogenic potential of HUVECs *in vitro* compared to each drug alone. Further, to functionally target proangiogenic CXC-chemokine signaling *in vivo*, we used SB225002 to treat tumors that have progressed over continued sorafenib treatment. We found that tumor progression stabilizes in sorafenib-refractory tumors when they are co-treated with SB225002 and sorafenib. Interestingly, once sorafenib was withdrawn, SB225002 alone did not inhibit tumor growth, and tumors progressed even faster than sorafenib-resistant tumors. This dramatic increase in tumor growth could be due to a sudden boost in the VEGF pathways as a consequence of withdrawal of sorafenib treatment, ultimately leading to tumor progression. We observed increases in plasma VEGF levels and VEGFR protein expression in tumors of treatment-refractory animals (data not shown).

These findings are in accordance with the published literature on antiangiogenic therapies. For example, in sorafenib-resistant HepG2 cell lines, significant increased proliferation was observed after withdrawal of sorafenib from the medium [47]. Similarly, rapid vascular regrowth has been observed after discontinuation of axitinib (AG013736) in mouse lung tumors [48] and discontinuation of sunitinib in patients with renal cell carcinoma [49]. Further, results from a large observational cohort study (BRiTE) also suggest that continued suppression of the VEGF pathway may be important to maximize bevacizumab’s clinical benefits in colorectal cancer treatment [50]. Data from our study indicate that treatment with SB225002 alone is insufficient. A combination of SB225002 and sorafenib is required to suppress tumor progression after anti-VEGF resistance in ovarian cancer xenografts. Thus, our findings indicate a risk of rebounding tumor growth after discontinuing sorafenib treatment in sorafenib-resistant SKOV-3 xenograft mice. Our results further support the hypothesis that continued suppression of the VEGF pathway may be important to maximize the benefits of antiangiogenic therapy in ovarian cancer treatment. At this time, we do not know whether the combined therapeutic benefit from CXCR2 inhibition is limited to sorafenib, or broadly applicable to other VEGFR inhibitors. Further studies will be needed to identify combination partners for maximal benefits among various CXCR2 and VEGFR2 inhibitors, using an orthotopic and patient-tumor derived xenograft models of ovarian cancer.

In summary, our findings reveal the CXCR2-mediated chemokine axis as potential pathways conferring tumor progression from continued treatment with sorafenib in ovarian tumors. Simultaneous targeting the chemokine receptor CXCR2 in combination with sorafenib is significant in improving the therapeutic benefit of sorafenib. To the best of our knowledge, this is the first report demonstrating a synergistic combined effect of CXCR2 inhibitor and sorafenib in the treatment of ovarian cancer. Several small molecule CXCR2 inhibitors with appropriate *in vivo* pharmacokinetic properties are currently in preclinical and clinical development for the treatment of cancer and inflammatory diseases [51–54]. Our findings merit further investigation to find the optimal dose and treatment schedule, and to determine the toxicity of this combination.

## Supporting Information

S1 Fig Concentration-effect curves of sorafenib and the combination of sorafenib and SB225002 in ovarian cancer cells and HUVECs. The top concentration of the combination was prepared by mixing the two drugs at a 1:2 ratio of their IC_50_ for ovarian cancer cells and a 1:1 ratio for HUVECs. Serial dilution (3x) was carried out from the top concentration to obtain a total of five dilutions (*n* = 4 replicates).
(TIF)

S1 File Supporting methods. (DOCX)

S1 Table IC_50_ values of sorafenib and SB225002 in ovarian cancer cell lines and HUVECs determined by MTS assay. (TIF)
