# Supplementary figures and images for: CXCR2 Inhibition Combined with Sorafenib Improved Antitumor and Antiangiogenic Response in Preclinical Models of Ovarian Cancer
Source: PLoS One. 2015 Sep 28;10(9):e0139237. doi: 10.1371/journal.pone.0139237 (PMC4587670; doi:10.1371/journal.pone.0139237)

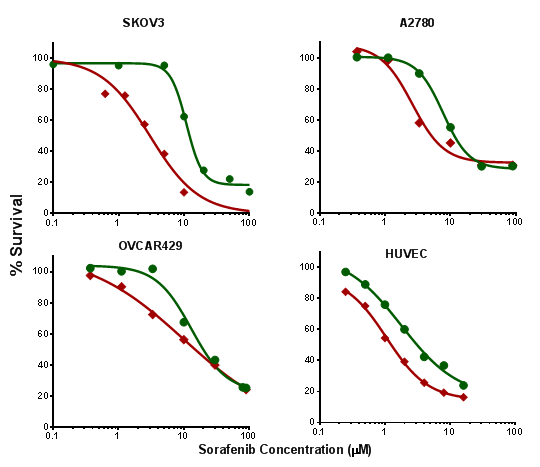

Supplement: S1 Fig — The top concentration of the combination was prepared by mixing the two drugs at a 1:2 ratio of their IC50 for ovarian cancer cells and a 1:1 ratio for HUVECs. Serial dilution (3x) was carried out from the top concentration to obtain a total of five dilutions (n = 4 replicates). (TIF) [file pone.0139237.s001.tif]

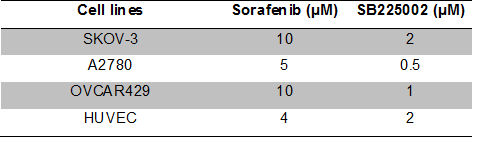

Supplement: S1 Table — (TIF) [file pone.0139237.s003.tif]
