# Supplementary material for: CXCR2 Inhibition Combined with Sorafenib Improved Antitumor and Antiangiogenic Response in Preclinical Models of Ovarian Cancer
Source: PLoS One. 2015 Sep 28;10(9):e0139237. doi: 10.1371/journal.pone.0139237 (PMC4587670; doi:10.1371/journal.pone.0139237)
Supplement: S1 File — (DOCX) [file pone.0139237.s002.docx]

**Supporting Methods**

Immunohistochemistry (IHC)

Immunohistochemical staining was performed on 5-µm formalin-fixed, paraffin-embedded serial mouse xenograft tissue sections. We incubated the sections overnight at 4ºC with primary antibodies to CD-31 (1:2000; rabbit polyclonal), Hif-1α (1:200; rabbit polyclonal), Ki-67 (1:200; rabbit monoclonal), and CXCR2 (1:200; rabbit polyclonal), followed by a 30 min incubation with a peroxidase-conjugated secondary antibody using the Vector Impress kit (Vector Laboratories, Burlingame, CA). Quantification of positively stained regions in slides was performed using the RGB image profiling method with ImageJ software. Each quantified value represents an average of three different sample measurements.

List of analysts used for angiogenesis array

MILLIPLEX MAP Human Angiogenesis/Growth Factor Magnetic Bead Panel, HAGP1MAG-12K: Angiopoietin-2, BMP-9, EGF, Endoglin, Endothelin-1, FGF-1, FGF-2, Follistatin, G-CSF, HB-EGF, HGF, IL-8, Leptin, PLGF, VEGF-A, VEGF-C, VEGF-D.

MILLIPLEX MAP Mouse Angiogenesis/Growth Factor Magnetic Bead Panel, MAGPMAG-24K: Amphiregulin, Angiopoietin-2, Betacellulin, EGF, Endoglin, Endothelin-1, FGF-2, Follistatin, G-CSF, HGF, IL-17A, IL-1β, IL-6, KC/CXCL1, Leptin, MCP-1, MIP-1α, PLGF-2, Prolactin, SDF-1, TNFα, VEGF-A, VEGF-C, VEGF-D, sALK-1, sCD31/PECAM-1, sFasL.

Expression of ELR+ CXC chemokines Gene expression

Transcriptome sequencing was performed using the Illumina MiSeq sequencer and Illumina TruSeq RNA v2 sample preparation kit and protocols (Illumina, Inc.). Before library construction, 500 ng of Total RNA was analyzed on the Agilent 2100 Bioanalyzer (Agilent Technologies, Inc.) using an RNA Nano Total RNA chip. We used 1 microgram of Total RNA per sample for library construction, per Illumina’s TruSeq RNA protocols. The quality of the resulting libraries and average insert size were determined using the Agilent 2100 Bioanalyzer (Agilent Technologies, Inc.) on a high sensitivity DNA chip. Samples were tagged with unique 6 bp indexes for multiplex sequencing, and equimolar amounts of three whole transcriptome samples were pooled together so that 6pM of the pool was run on the Illumina MiSeq sequencer using paired-end 2x150-bp sequencing. Before loading, we spiked 5% phiX library into the library pool for quality control purposes, per Illumina’s recommendations. Approximately 30,000,000 reads total were collected for each run. Analysis was performed using Perkin Elmer’s Genesifter software and alignment to the *H. sapiens* and *M. musculus* reference genomes.
